# Supplementary material for: Short Pyridine-Furan Springs Exhibit Bistable Dynamics of Duffing Oscillators
Source: Nanomaterials (Basel). 2021 Nov 30;11(12):3264. doi: 10.3390/nano11123264 (PMC8707925; doi:10.3390/nano11123264)
Supplement: Supplementary file 1 [file nanomaterials-11-03264-s001.zip › nanomaterials-1477470-supplementary-final.pdf]

Supporting Information: Short Pyridine-Furan Springs Exhibit Bistable Dynamics of Duffing Oscillators

Vladik A. Avetisov,* Maria A. Frolkina, Anastasia A. Markina,* Alexander D. Muratov, and Vladislav S. Petrovskii

N. N. Semenov Federal Research Center of Chemical Physics, Russian Academy of Sciences, Kosygina 4, 119991 Moscow, Russia

E-mail: avetisov@chph.ras.ru; markina@mpip-mainz.mpg.de

Simulation protocol

Parameters for Molecular Dynamics simulation

Morphology simulations were performed using the Gromacs2019[S1] simulation package. Lennard–Jones parameters were taken from the OPLS-AA[S2] force field with a scaling factor of 0.5 for the 1–4 interactions. The SPC/E model[S3] was used for water. Long-range electrostatic interactions were treated using a smooth particle mesh Ewald technique[S4] with a cut-off of 1.2 nm. Bond vibrations were constrained with a LINCS[S5] algorithm. All calculations were performed in the NVT ensemble using the canonical velocity-rescaling thermostat, as implemented in the Gromacs2019 simulation package.

The parameterization of oligo-PF springs is presented in Figure S1a-b. The OPLS-AA force field contains parameters only for single pyridine and furan molecules. First, $C4$ and $C6$

atoms have a covalent bond instead of hydrogen atoms and the partial charge of hydrogens were added to carbon atoms to maintain the neutrality. All the other partial charges are taken from OPLS-AA force-field. Second, there is only one undefined angle bond for atoms $C4-C6-N11$, which is $CW-CA-NC$ angle type in OPLS-AA. We used $CA-CA-NC$ angle due to similarity of CA and CW types. Both correspond to aromatic $sp2$-hybridized carbon.

The simulation was started from a random initial configuration. An equilibrated state were reached in two steps. First, a short run of 10 ps was carried out in the NVT ensemble with a time step of 0.01 fs with the leapfrog integrator for motion equations. Then the second part of equilibration was done for another 10 ns with a time step of 2 fs. After the equilibration, a long trajectory of 500 ns is obtained to achieve a clear picture of transitional behaviour.

To simulate the behaviour of the oligo-PF5 under the external load, one end of it was fixed and the other end was pulled by an external force (see Figure S1c). We used a simulation box of size $7 \times 7 \times 7\ nm^3$ due to technical aspects of Gromacs pulling algorithm. The axis of an initial conformation of oligo-PF5 was oriented in the XY plane. The longitudinal load F was applied along Z-direction to the center of mass of the last monomer unit and directed toward the attraction point, which was located along the vector connecting the left and right ends of the molecule.

At the same time, to simulate behaviour of the oligo-PF7, both ends of the molecule in the stressed state were fixed in a simulation box of size $3.7 \times 3.7 \times 3.7\ nm^3$. The different distances P were obtained by pulling one end of the oligo-PF7. Then the pulled end was fixed and the vibrations were explored with different distances P. Various initial structures are shown in Figure S2.

In both cases, the center of mass of the first monomeric unit was fixed using a spring potential of $k = 100\,\mathrm{kJ} \cdot \mathrm{mol}^{-1} \cdot \mathrm{nm}^{-2}$; other specific constraints for bond length or atom positions were applied.

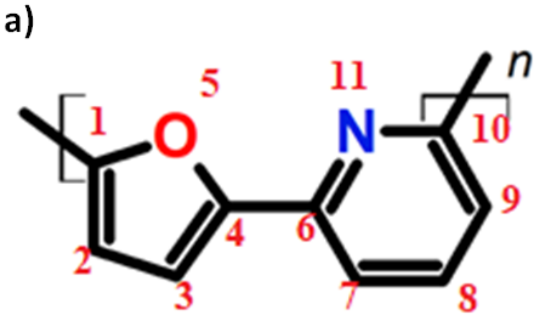

b)

Atom number	OPLS-AA type	Partial charge
C1, C4	opls_567	+0.123
C2, C3	opls_568	-0.154
O5	opls_566	-0.190
C6, C10	opls_521	+0.485
C7, C9	opls_522	-0.447
C8	opls_523	+0.227
N11	opls_520	-0.678
H7, H9	opls_525	+0.155
H8	opls_526	+0.065
H2, H3	opls_570	+0.126
H-term. pyridine	opls_524	+0.012
H-term. furan	opls_569	+0.142

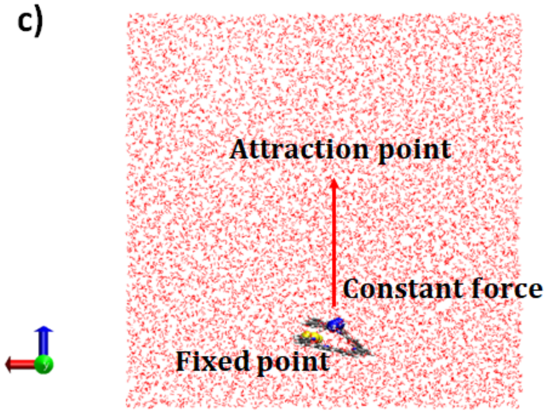

Figure S1: (a) The chemical structure of a PF monomer, (b)parameterization of PF oligomers in OPLS-AA force field types and partial charges, (c) a scheme of the simulation box for the oligo-PF5 system.

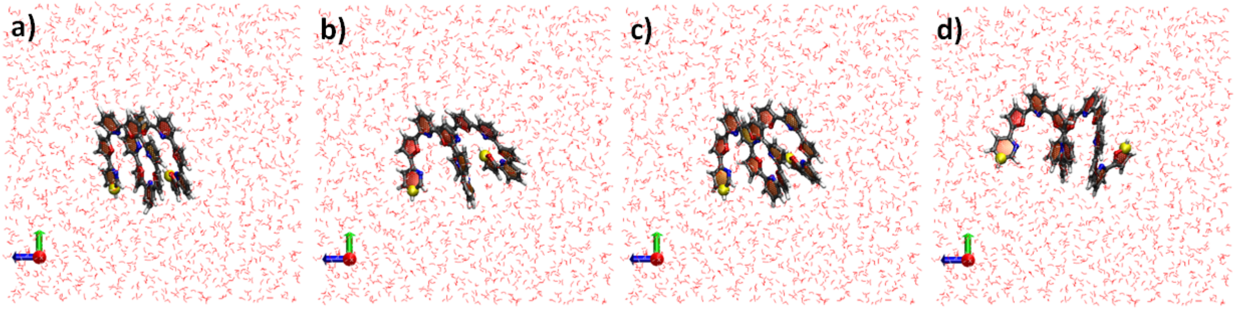

Figure S2: Initial states of the oligo-PF7 are shown with different distances P = (a) 0.74, (b) 1.07, (c) 1.03, and (d) 1.62 nm between fixed points (yellow dots).

Oligo-PF5 in different solvents

In order to find out the impact of water on the dynamics of oligo-PF, we also model its stretching in hydrophobic solvent (tetrahydrofuran (THF)) and vacuum. The simulation protocol in both cases is similar to the one described in the section Parameters for Molecular

Dynamics simulation above. One end of the oligo-PF5 was fixed and the pulling force was applied to another end.

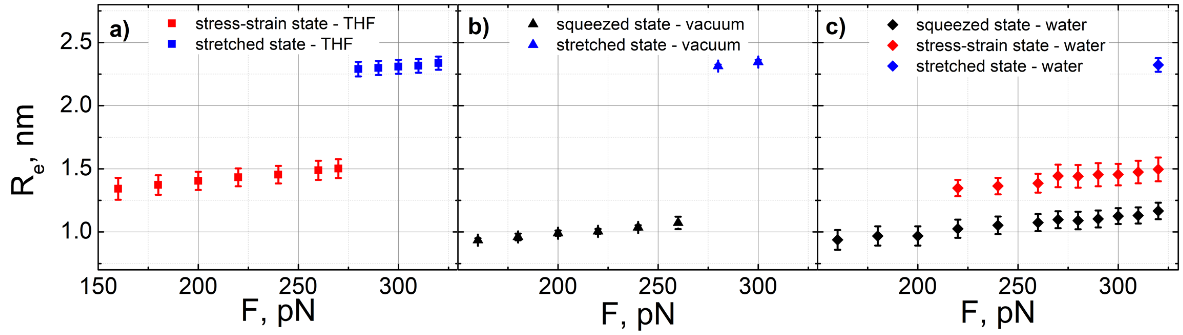

Figure S3: The state diagram of the oligo-PF5 in different solvents. (a) The diagram of the oligo-PF5 states in THF, (b) vacuum and (c) water.

The end-to-end (R_{ee}) distance both in THF and vacuum gradually increases along with the force up to the value of $F = 275\,\text{pN}$ at which point the oligo-PF5 helix turns to the fully stretched state (see Figure S3a and S3b). Note that the oligo-PF5 in THF is present in the stress-strain at the pulling forces below $F = 275\,\text{pN}$ due to the hydrophobic nature of the solvent.

Cis- and trans- isomerization of oligo-PF

Following the quantum mechanical calculations of Harikrishna Sahu and Panda[S6], we used the oligo-PF molecule in the cis-conformation, but while applying the supercritical load exceeding 330 pN we observe the transition of the overstretched oligo-PF5 to the trans-conformation. Without any pulling, the oligo-PF molecule stays in cis-conformation and no additional torsion potentials were applied except default OPLS-AA parameters.

Parameters for periodic signal

Stochastic resonance was obtained by applying a periodic signal leading to the swinging of the oligo-PF bistable potential. An oscillating force was implemented by setting a charge

(+1) on the oligomer, adding a compensating charge (–1) as a counter ion in the solvent and applying a periodical electric field. The additional charge was placed on the end group of the oligo-PF5 and spread between the first, second and fifth atoms of the furan group (see Fig. S1a). In case of the oligo-PF7, the charge is set on the middle fourth monomer unit and spread between the seventh, eighth and ninth atoms of the pyrydine group. In all cases the additional chargers are equally distributed between the chosen atoms.

The periodic field in the Gromacs2019 package is defined by an equation

$$E(t) = E_0 \exp\left[-\frac{(t-t_0)^2}{2\sigma^2}\right] \cos\omega(t-t_0),$$

where the exponential part modulates the periodic part with the pulsing behaviour, E_0 is the amplitude of the signal and ωt_0 is the oscillation phase. Here we use only the static part when $\sigma = 0$ along z-axes (see Figure S1c and Figure S2). An external oscillating electrical field was directed along the constant force direction in the oligo-PF5 case. In the oligo-PF7 system, the periodic signal was applied along a line connecting fixed atoms.

Results

Spontaneous vibrations data

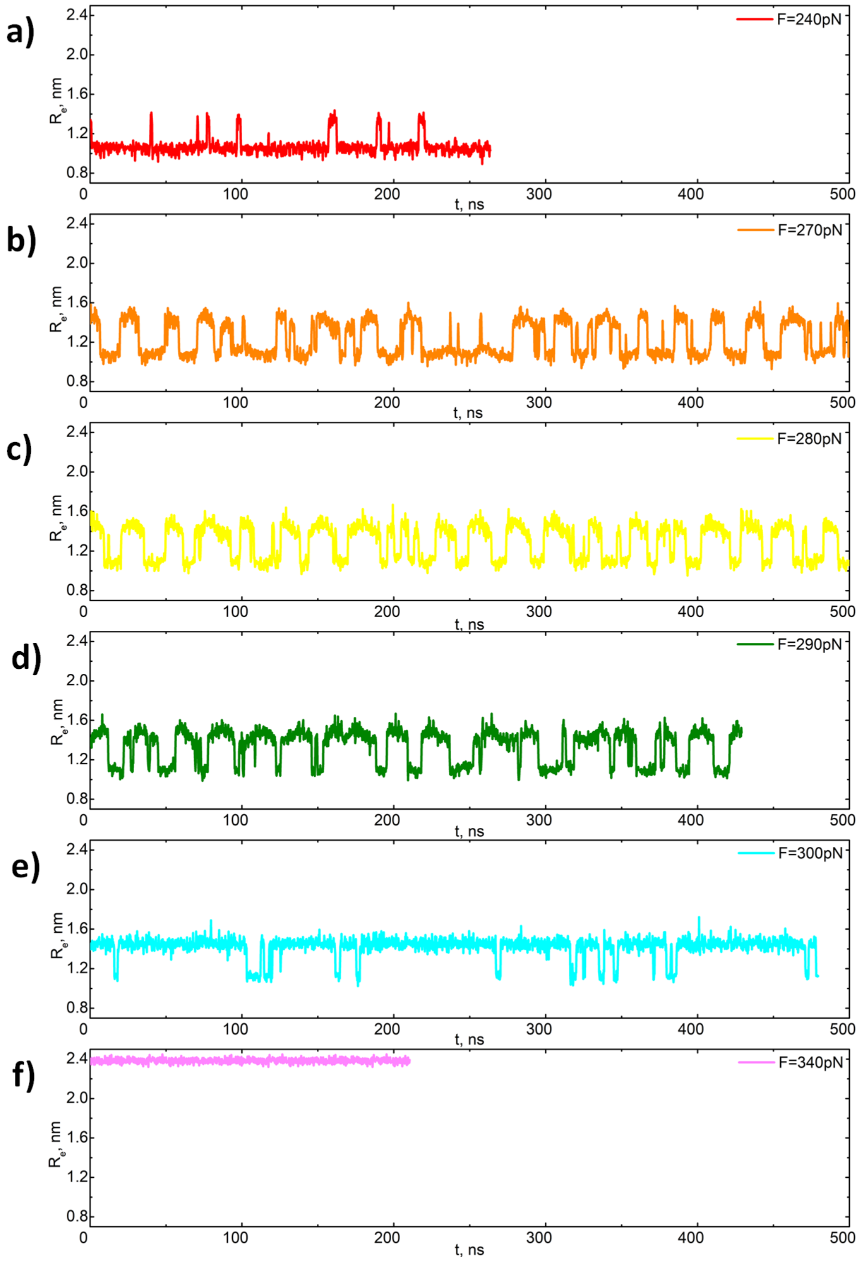

Figure S4: a-f)Trajectories of spontaneous vibration with different stretching forces. One can see that the oligo-PF5 moves from the close state to the open state with the increasing of the force F.

The trajectories of the spontaneous vibrations are shown in Figure S4. These trajectories are used to calculate the state distribution, which is shown in Figure S6. Optimal conditions for stochastic resonance are obtained from these trajectories. The main condition for clear resonance is a symmetrical distribution of the oligo-PF5 states. Under the super-critical load (higher than 320 pN (Figure S4f)), the oligo-PF5 appears to be in a fully stretched state and is not able to return to the stress-strain state. In the fully stretched state slow transitions from cis-state to trans-state occur and block the sharp transition back.

Mean lifetime estimation

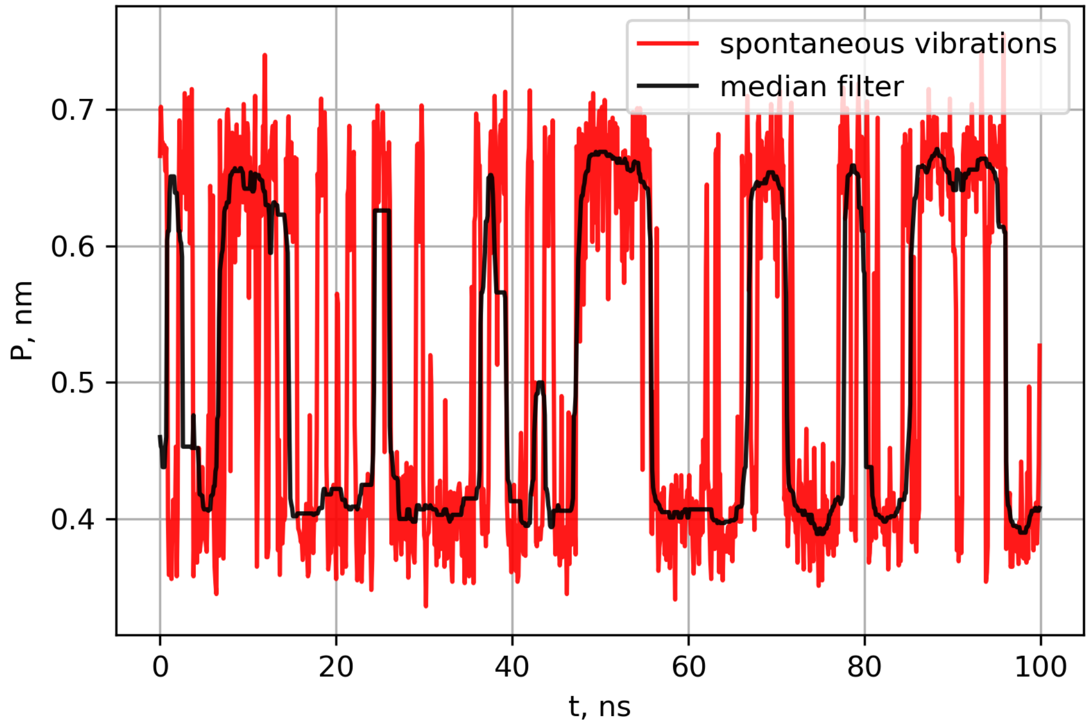

Figure S5: The spontaneous vibrations trajectory with and without the median filter usage.

In the oligo-PF systems, one can observe the dynamics at two temporal scales: local and global. The first one is characterized by fast fluctuations near the left-end/right-end states in case of oligo-PF7 or squeezed/stress-strain states in case of oligo-PF5. However, the

global dynamics (the switching between the ends) is more important for stochastic resonance. To calculate the mean lifetime, the local dynamics were excluded using the median filter $ndimage.median\_filter$ from an open-source software SciPy for Python 3 with a median filter window size of 3.5 ns, which corresponds to local vibrations. The resulting trajectory is shown in S5. According to the estimation, the mean lifetime of oligo-PF7 spring was $\tau = 6.5$ ns for the most symmetric distribution at the distance $D = 1.03$ nm (see Figure 4d). The similar mean lifetime for oligo-PF5 was $\tau = 6.14$ ns and corresponded to the external force $F = 279$ pN.

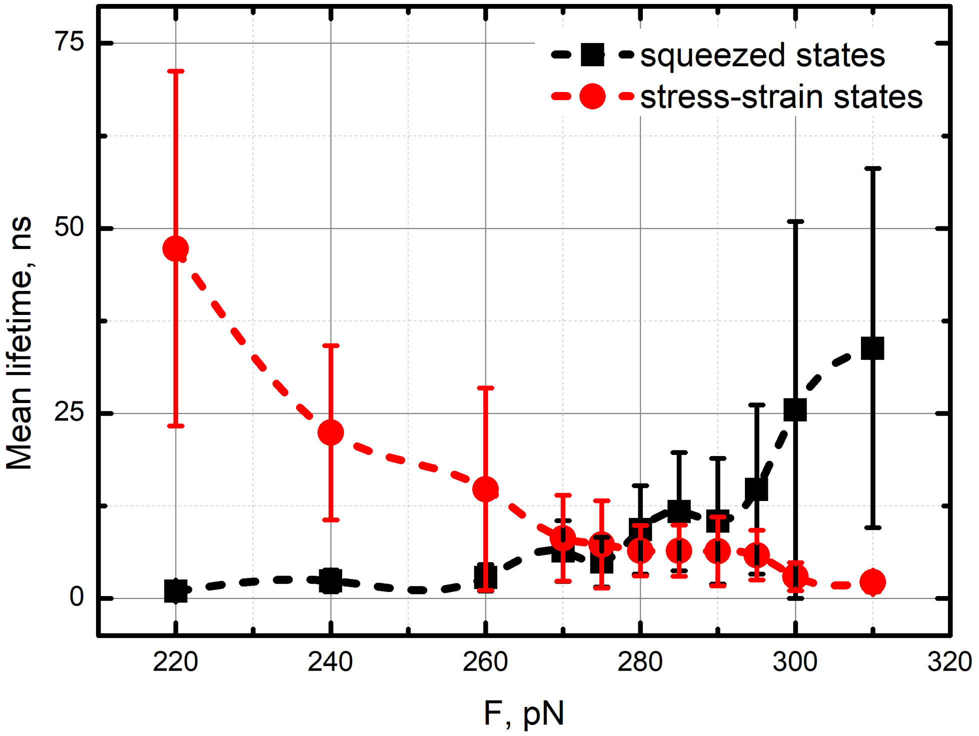

Figure S6: Mean lifetimes for squeezed (black curve) and stress-strain (red curve) states for the oligo-PF5 are shown.

State diagram of oligo-PPF7

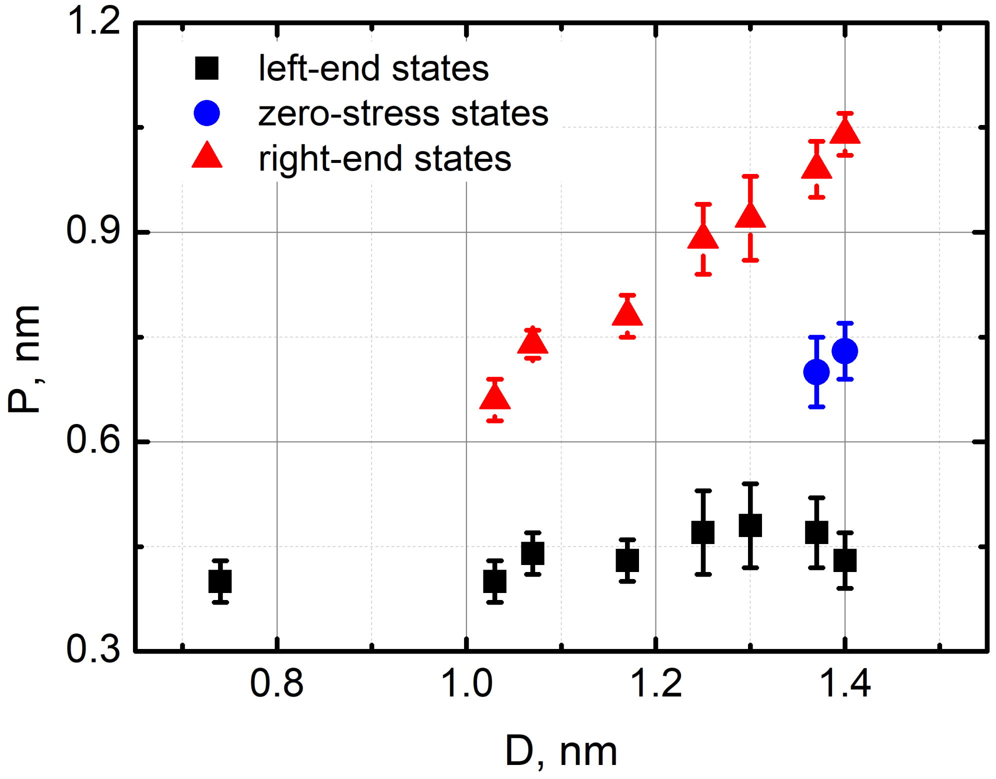

Figure S7: The state diagram of oligo-PF7 states.

The state diagram of the oligo-PF7 states with $D > 1.30$ nm is shown in S7. The wide repulsive zone of the zero-stress states becomes the third attractive state after the threshold of ~ 4 stacking lengths.

Signal-to-noise ratio in resonance

To analyse the stochastic resonance data, power spectra $S(\nu)$ for each trajectory of oligo-PF springs were calculated, which are shown in the Figure 3b and 5b. The power spectrum is defined as a Fourier transform of an auto-correlation function,

$$S(\nu) = \int_{-\infty}^{\infty} \langle Z(t)Z(t-\tau)\rangle e^{-2\pi i\nu\tau} d\tau, \tag{1}$$

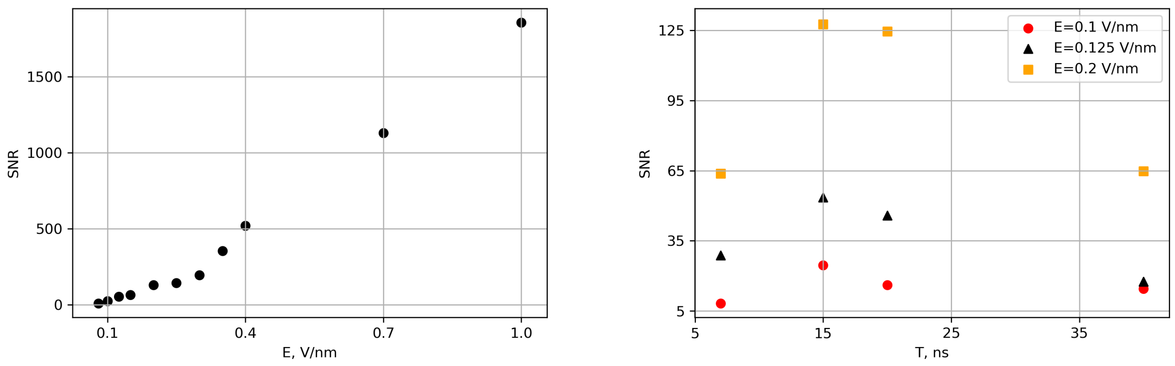

(a) (b)

Figure S8: The signal-to-noise ratio. (a) The dependence of the SNR on the amplitude E; (b) The dependencies of the SNR on the external oscillation field period with amplitude E =0.1, 0.125 and 0.2 V/nm.

where $\langle Z(t)Z(t-\tau)\rangle$ is the normalized auto-correlation function, since $Z(t) = z(t)/\|z(t)\|$ is the normalised signal of the standard score $z(t)$ for the oligo-PF5 resonance trajectory R_e (or P in the oligo-PF7 case).

The signal-to-noise ratio (SNR) is a standard characteristic of the stochastic resonance. We define SNR as a ratio of the main resonance peak amplitude from the power spectrum of stochastic resonance to the mean noise value near the peak. The dependence of the SNR on the amplitude E is shown on S8a. The values could be approximated by two linear series that that intersect at $E = 0.3$ V/nm, which could mean a transition from stochastic resonance to forced oscillations.

The dependencies of the SNR on the period of oscillating field for different amplitudes E are shown on S8b. The main SNR peak is observed close to the doubled mean lifetime of the state in the spontaneous vibration mode.

References

(S1) Abraham, M. J.; Murtola, T.; Schulz, R.; Páll, S.; Smith, J. C.; Hess, B.; Lindahl, E. GROMACS: High performance molecular simulations through multi-level parallelism

from laptops to supercomputers. *SoftwareX* **2015**, *1*, 19–25.

(S2) Kaminski, G. A.; Friesner, R. A.; Tirado-Rives, J.; Jorgensen, W. L. Evaluation and reparametrization of the OPLS-AA force field for proteins via comparison with accurate quantum chemical calculations on peptides. *The Journal of Physical Chemistry B* **2001**, *105*, 6474–6487.

(S3) Berendsen, H.; Grigera, J.; Straatsma, T. The missing term in effective pair potentials. *Journal of Physical Chemistry* **1987**, *91*, 6269–6271.

(S4) Essmann, U.; Perera, L.; Berkowitz, M. L. A smooth particle mesh Ewald method. *The Journal of chemical physics* **1995**, *103*, 8577–8593.

(S5) Berk, H. P-LINCS: A parallel linear constraint solver for molecular simulation. *Journal of Chemical Theory and Computation* **2008**, *4*, 116–122.

(S6) Harikrishna Sahu, P. G., Shashwat Gupta; Panda, A. N. Structure and optoelectronic properties of helical pyridine–furan, pyridine–pyrrole and pyridine–thiophene oligomers. *Physical Chemistry Chemical Physics* **2015**, *17*, 20647–20657.
